# Effects of Traffic Vibrations on the Flexural Properties of Newly Placed PVA-ECC Bridge Repairs

**DOI:** 10.3390/ma12203337

**Published:** 2019-10-13

**Authors:** Xiaodong Zhang, Shuguang Liu, Changwang Yan, Xiaoxiao Wang, Huiwen Wang

**Affiliations:** 1 School of Materials Science and Engineering, Inner Mongolia University of Technology, Hohhot 010051, China; zhangxd8808@126.com; 2 School of Mining and Technology, Inner Mongolia University of Technology, Hohhot 010051, China; yancw20031013@126.com; 3 Beijing Tuowei Times Architectural Design Co., Ltd., Beijing 100020, China; wanghw0717@126.com

**Keywords:** PVA-ECCs, bridge repairs, setting periods, traffic vibrations, strain-hardening characteristics, flexural properties

## Abstract

Polyvinyl alcohol fiber reinforced engineering cementitious composites (PVA-ECCs) exhibit excellent tight-cracking and super-high toughness behaviors and have been widely used in bridge repair projects. In reality, the conventional method in bridge repair is that a portion of the bridge is closed and repaired while the other portion is left open to traffic. Consequently, newly placed PVA-ECC bridge repairs (NP-ECC-BRs) are exposed to continuous traffic vibrations (TRVs), even during the setting periods. However, whether or not TRVs affect the expected flexural properties of NP-ECC-BRs remains unknown. The purpose of this investigation was to determine the effects of TRVs on the attainable flexural properties of NP-ECC-BRs. For this purpose, a total of 324 newly fabricated thin-plate specimens were exposed to different vibration variables using self-designed vibration equipment. After vibration, a four-point flexural test was conducted to determine the flexural properties of the specimens. The results indicate that the effects of TRVs on the strengths of NP-ECC-BRs was significantly negative, but insignificantly positive for flexural deformation. We concluded that in the design of PVA-ECC bridge repairs, effects of TRVs on the flexural deformation capacity of NP-ECC-BRs are not a cause for concern, but serious consideration should be given to the associated reduction of flexural load-bearing capacity.

## 1. Introduction

Concrete was the most-used solid material over the past century. To date, a huge number of concrete structures have needed repair, for which enormous sums are spent annually [1,2,3]. Polyvinyl alcohol fiber reinforced engineering cementitious composites (PVA-ECCs) possess unique tight-cracking, strain-hardening, and super-high toughness behaviors under flexural loading [4], hence are becoming an attractive choice for the repair of existing concrete structures. Recently, PVA-ECCs have been widely used to repair concrete bridge deck slabs [5], pavements [6], overlays [7], and expansion or non-expansion joints [8].

In reality, completely closing traffic during bridge repair is unrealistic, so the conventional method is that a portion of the bridge is closed and repaired while the other portion is left open to traffic. As a result, newly placed PVA-ECC bridge repairs (NP-ECC-BRs) are exposed to continuous traffic vibrations (TRVs), even during the curing times. However, whether or not TRVs affect the expected flexural properties of NP-ECC-BRs remains unknown. 

Similar to ordinary concrete and other cement-based composites bridge repairs, NP-ECC-BRs feature different rheological characteristics [9] during different setting periods and thus present variable physicochemical states. Therefore, it could be speculated that NP-ECC-BRs are vulnerable and sensitive to TRVs, and a series of their physicochemical processes might be affected during different setting periods.

Before the initial set, continuous TRVs will cause some extent of bleeding to the cement motor or concrete mixture [10], and this might be the same for PVA-ECCs under TRVs. During the setting period (between the initial and final set), low-density calcium silicate hydrate (C-S-H) gels in the cement matrix are gradually transferred to high-density C-S-H gels and agglomerate into larger C-S-H particles [11,12]. If TRVs occur during the setting period, this will probably damage or obstruct the connection of the solid skeleton [13] in the PVA-ECCs, as well as the bond of C-S-H particles or even that of C-S-H gels. After the final set, the hydration of cement is mainly restricted by the diffusion of capillary water in the matrix [14,15], any TRVs that occurred during this period would probably result in the accelerated transportation of free water from the capillary pores toward the interface of the anhydrous cement grains, which would be convenient for the consumption of anhydrous cement grains [16] and further increase the degree of hydration of the matrix, thereby affecting the flexural properties of the PVA-ECCs as a consequence.

As there is the potential for TRVs to greatly affect the properties of newly placed bridge repairs, a number of related investigations have been carried out over recent decades. Previous studies mostly focused on newly placed concrete bridge repairs (NP-C-BRs) as the research object, taking the compressive and bond strength, or splitting tensile strength of NP-C-BRs as vibration responses. The majority of these studies showed that the effects of TRVs on the strengths of NP-C-BRs should not be a cause for concern or need to be considered as a serious risk during bridge repair or widening [17,18,19,20,21]. Through a large number of experimental and field investigations, Manning (1981) pointed out in a research report for American association of state highway and transportation officials (AASHTO) that TRVs seem have no substantial effects on the compressive and bond strengths for NP-C-BRs. He also emphasized that both bond and compressive strengths even appeared to increase slightly for NP-C-BRs of high quality and low slump and, in that case, traffic could be maintained on bridge decks undergoing repair [17]. Subsequently, similar conclusions were drawn by Harsh (1986) [18].

Many recent studies [19,20,21] have also shown that the effects of TRVs on both the compressive and bond strengths of NP-C-BRs are not a cause for concern. Weathere [19] performed an experimental test to simulate the effects of staged bridge deck construction on the bond strength of concrete/reinforcing steel; the results indicate that after being imposed to different displacements, the bond strength of concrete/reinforcing steel of NP-C-BRs was still capable of developing the actual yield strength of the reinforcing bars. Wang [20] performed an experimental test to study the effects of TRVs on the compressive strength of newly placed high performance concrete (HPC) repairs; the results show that after being vibrated under the imposed vibration model of 2 Hz–3 mm and 4 Hz–3 mm, the 28 days’ compressive strength of HPC bridge repairs decreased slightly by 3% (which could be ignored). Hong [21] conducted laboratory studies and a field test to investigate the effect of TRVs on the compressive and bond strengths of fresh concrete during bridge widening; the results show that the effect of TRVs on the strengths of NP-C-BRs should not require serious consideration if the durations of vibration are within 6 h and the corresponding peak particle velocities (PPVs) are within 0.3 cm/s.

Some current studies have shown that TRVs not only significantly reduce the compressive and bond strengths, but also result in a considerable reduction in the tensile strength, flexural strength, and elastic coefficient [22,23,24] of NP-C-BRs. Zhang [22] observed apparent macrocracks on the surface of NP-C-BRs and, moreover, detected serious internal damage after the specimens were vibrated during the setting period (the period between the initial and final set, where the penetration resistance was within the scope of 3.5 to 28 MPa). As this special period was the most vulnerable to TRVs, Zhang [22] described it as a vibration-sensitive stage. Kwan [23] and Ng [24] investigated the effects of TRVs on curing concrete stitch and found that if vibrations began right after pouring, relatively small cracks or slackness caused by threshold curvature would result in a significant reduction (above 20% reduction) in bond and contraflexural strength.

Additionally, over the years, a number of studies have been performed experimentally, methodologically, and numerically to reveal the performance of newly designed concrete structure or newly placed concrete materials subjected to various sources of dynamic loads, including seismic [25,26], pile driving [27], and blasting [28], and constructive research progress has been achieved.

So far, PVA-ECCs appear to have a bright future in the repair and retrofitting of existing constructed facilities, having been widely used especially in maintenance of the upper structures of bridges. Nonetheless, despite the great potential for TRVs to affect the properties of NP-ECC-BRs, to the best of our knowledge, we found that few of the previous studies have revealed the effects of TRVs on the properties of NP-ECC-BRs, except for the authors’ previous work that revealed the effects of TRVs on the tensile behaviors of NP-ECC-BRs [29].

The purpose of this study was to study the effects of TRVs on the flexural properties of NP-ECC-BRs. For this purpose, self-designed vibration equipment was adopted to simulate TRVs, and a total of 333 (37 groups of nine) thin-plate PVA-ECC specimens with size of 400 × 100 × 15 mm were fabricated. Each group of thin-plate PVA-ECC specimens was vibrated under three designed variables, including the age at which the specimens were vibrated (AWV), duration of vibration (DV), and vibration frequency. Finally, after being cured for 28 days, all 37 groups of specimens were tested with a four-point flexural test method to determine their flexural properties.

## 2. Materials and Methods

### 2.1. Materials 

Materials consisting of ordinary Portland cement, silica sand, fly ash, water, and polyvinyl alcohol (PVA) fiber were used. Additives consisting of viscosity modifying agent (VMA), high-efficiency defoamer (HED), and high-efficiency water reducing agent (HEWRA) were used to modify the properties of the cement matrix. Detailed source information of the materials is listed in Table 1**.** The basic physical properties of ordinary Portland cement are listed in Table 2, and the chemical compositions of Portland cement are listed in Table 3. The chemical compositions of fly ash with particle sizes of 0.5–2.0 μm are listed in Table 4. The physical properties of the PVA fiber are listed in Table 5. The mixture proportions of the materials are listed in Table 6.

The particle sizes of high-quality silicon sand ranged from 75 to 135 μm. The water-reducing efficiency of PCSP was about 33.0% (provided by the manufacturer). The effects of the VMA were to increase the cohesiveness and water retention of the matrix, as well as to prevent fiber from aggregation. The effects of the HED were to inhibit or eliminate the production and propagation of air in the mixture during mixing. 

In this investigation, a constant PVA fiber volume fraction of 2.0% and a constant water-to-binder ratio of 0.24 were used.

### 2.2. Methods

#### 2.2.1. Specimen Preparation and Testing Procedure

First, a penetration test was conducted, and the setting times of PVA-ECC mixtures were obtained for the predetermination of vibration variables. Second, a total of 333 (37 groups of nine) thin-plate PVA-ECC specimens were fabricated to meet the vibration variables. The 37 groups of specimens included one group of control specimens that would not be vibrated, and 36 groups of specimens that would be vibrated under different vibration variables. Third, the 36 groups of specimens were vibrated using self-designed vibration equipment under different vibration variables so that the simulation of TRVs was achieved. Fourth, after vibration, all 37 groups of specimens were cured for 28 days in a few sealable boxes in which a layer of water was reserved, and a stainless-steel frame was placed; the specimens were placed on the stainless-steel and not in contact with water. This curing method ensured that the relative humidity in the curing boxes was higher than 90% and the temperature in the curing boxes was approximately 20 ± 5 °C. Finally, after being cured for 28 days, all 37 groups of PVA-ECC specimens were tested using a four-point flexural test method.

#### 2.2.2. Vibration Equipment and Variables

**Figure 1:**
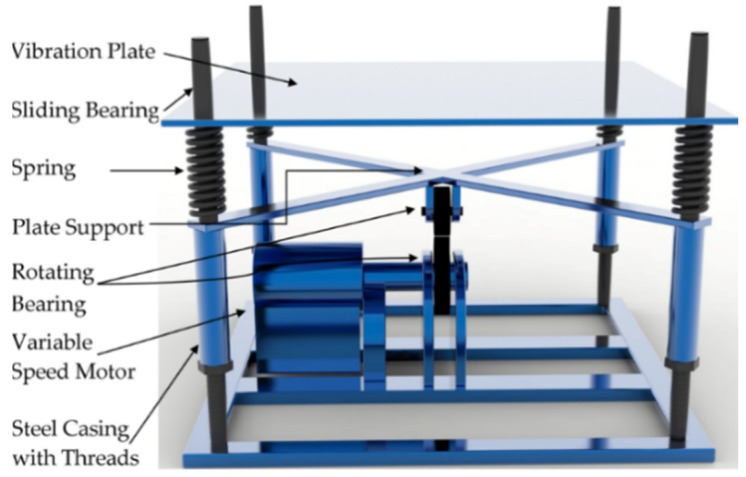
Self-designed vibration equipment [29].

TRVs were simulated using self-designed vibration equipment, as shown in Figure 1. The vibration table of the equipment where specimens were placed was driven by a motor underneath. The rotational frequencies of the motor were controlled by a frequency converter. The scope of vibration frequency of the working table could be adjusted from 1 to 10 Hz through the frequency converter. The vibration model of this equipment was designed to produce simple harmonic vibration. 

In this study, three variables, namely, age when the specimens were vibrated (AWV), magnitude of vibration frequency (VF), and duration of vibration (DV), were considered to be of greatest interest regarding their potential to affect the flexural properties of NP-ECC-BRs.

**Figure 2:**
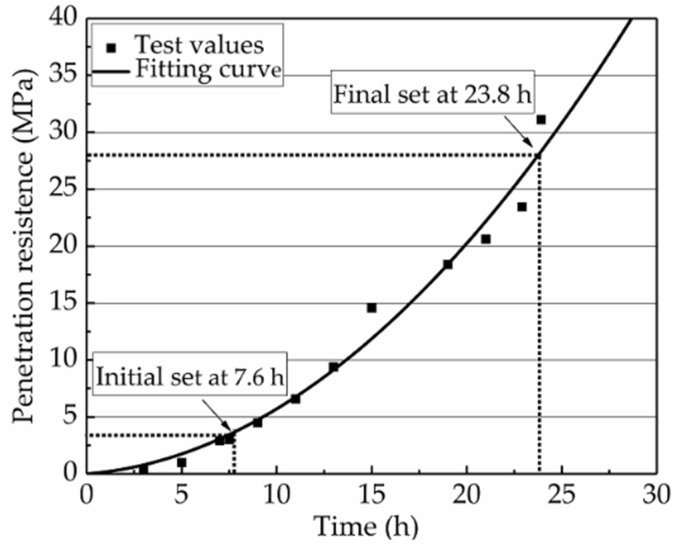
Results of the penetration test [29].

The first variable, AWV, the age or time when the specimens would be vibrated, was predetermined by conducting a penetration test according to the Chinese National Standard JTG E30–2005 [32]. According to this standard, the hours at which the cement or concrete mixtures reach the stages of the initial and final set are defined as when the penetration resistance reaches 3.5 and 28.0 MPa, respectively. The hours of the initial and final set of the PVA-ECCs in this study were obtained as 7.6 and 23.8 h, respectively, as shown in Figure 2. To investigate the effects of TRVs on the flexural properties of NP-ECC-BRs, the specimens were designed to be vibrated during three setting periods. The selected ages at which the specimens were vibrated, and their corresponding setting periods, are listed in Table 7.

The second variable, DV, was defined as the duration of continuous and uninterrupted vibration disturbance in this study. DVs of 2, 5, 8, and 11 h were chosen to explore whether and to what extent the length of vibration affects the flexural properties of NP-ECC-BRs.

The third variable, vibration frequency, was determined according to previous experimental and field studies on concrete bridges. The vibration frequency was selected to investigate the effects that different magnitudes of TRVs have on the flexural properties of NP-ECC-BRs. Jiang [33] conducted a large number of experimental investigations and field tests and obtained the vibration frequencies of different types of concrete bridges induced by moving vehicles, which ranged from 1.74 to 5.0 Hz, and the corresponding vibration amplitudes, which were in the range of 3.3–9.3 mm The Swiss Federal Laboratories for Materials Science and Technology (EMPA) [34] conducted a series of dynamic tests on 226 concrete girder bridges and obtained a regression empirical equation of the first fundamental frequency and bridge span, as shown in Equation (1).

(1) $$f=90.41^{-0.933}$$

where l is the span (m) of the concrete girder bridge and f is the first fundamental frequency (Hz). According to Equation (1), TRV frequencies of concrete bridges with the commonest spans of 60, 40, 30, and 20 m were calculated as 1.97, 2.88, 3.77, and 5.50 Hz, respectively. Based on these results, vibration frequencies of 2, 3, 4, and 5 Hz were selected in this study, and the corresponding amplitude was maintained as 5 mm according to Jiang [33]. These selected frequencies could cover most of the medium-span and mini-type concrete bridges which are the most common bridge types found.

#### 2.2.3. Flexural Test Program

A four-point flexural test method was adopted through an MTS exceed universal test system (MTS E43.104, Eden Prairie, MN, USA) with a range of 10 kN. The flexural test was performed on a model with an imposed displacement of 0.06 mm/s during loading. The mid-span deflection was measured by a linear variable differential transformer (LVDT). The loads were collected by a tension-compression sensor (BLR-1/1T, Donghua Electronics, Shanghai, China). The tested data were collected through a Static Strain Collection and Analysis System (DH3820, Huadong Test, Jinjiang, China).

**Figure 3:**
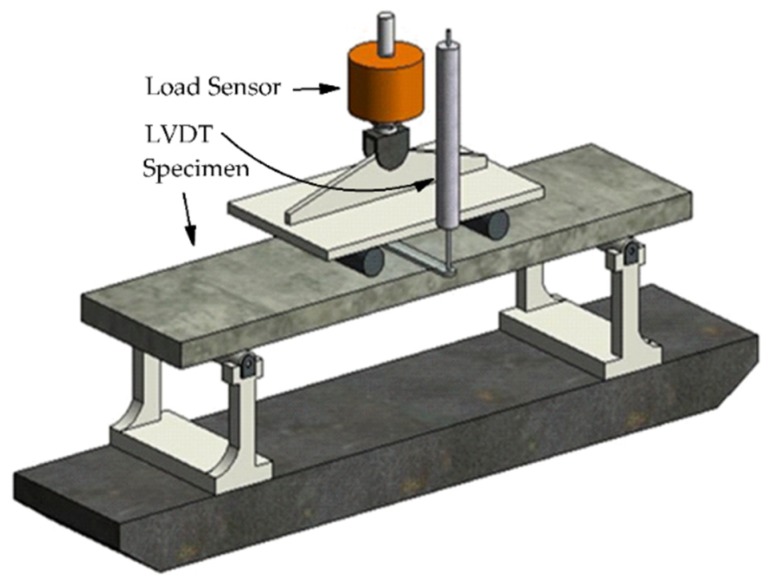
Sketch of the four-point flexural test.

The tested span and force arm of the specimens were designed as 300 and 100 mm, respectively. A sketch of the four-point flexural test is shown in Figure 3.

#### 2.2.4. Specimen Design

The size of the specimens was 400 mm × 100 mm × 15 mm. To meet the designed variables, 36 groups of newly poured PVA-ECC specimens were vibrated under operating conditions for (1) ages of 1.5, 8, 15, 23, 36, and 48 h under the combination of a duration of 5 h and imposed vibration frequencies of 2, 3, 4, and 5 Hz, referred to as Var. 1; and (2) durations of 2, 5, 8, and 11 h at ages of 8 h under imposed vibration frequencies of 2, 3, 4, and 5 Hz, referred to as Var. 2. The detailed numbers, variables, and operating conditions of all the 324 PVA-ECC specimens are shown in Table 8.

#### 2.2.5. Data Analysis

To minimize the variance of the data measured in this study, the winsorized mean of the measured data for each group of specimens was taken as the representative value of the corresponding group. Additionally, for the one specimen in each group whose maximum mid-span deflection was the closest to the winsorized mean of the group, its load versus mid-span deflection (P–δ) curve would be taken as the representative P–δ curve of its group.

## 3. Results and Discussion

The results of calculated winsorized means of the cracking load (P_c_) and the corresponding mid-span deflection (δ_c_), extreme flexural load (P_u_) and the corresponding mid-span deflection (δ_u_) of the specimens are shown in Table 9. 

**Figure 4:**
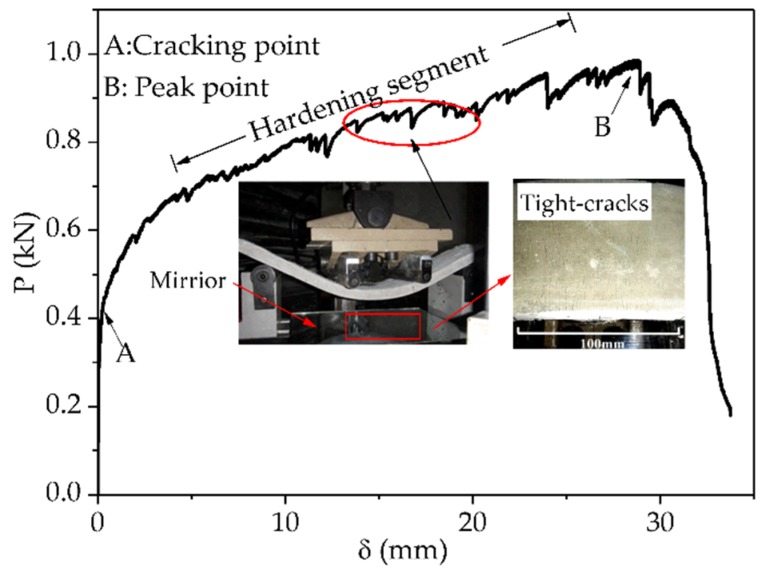
Representative load versus mid-span deflection (P–δ) curve of the control specimen, showing the classical strain-hardening and super-toughness characteristics of polyvinyl alcohol fiber reinforced engineering cementitious composites (PVA-ECCs).

Strictly speaking, it should be pointed out that P_c_ and δ_c_ correspond to the load and deflection when the first crack appeared on the specimen. In this study, the end point of the elasticity region in the P–δ curve was defined as the cracking point, as shown in Figure 4, point A. For convenience, the corresponding load and deflection at the cracking point were taken as the P_c_ and δ_c_ of the specimen. Similarly, the corresponding load and deflection at the peak point in the P–δ curve were taken as the P_u_ and δ_u_ of specimens, as shown in Figure 4, point B.

### 3.1. Effects of AWV on the Flexural Properties of NP-ECC-BRs

In this section, to determine the effects of AWV on the flexural properties of NP-ECC-BRs, a total of 216 (24 groups, and nine specimens in each group) thin-plate PVA-ECC specimens were tested by a four-point flexural test after the specimens were vibrated under the operating conditions for Var. 1. 

#### 3.1.1. Effects of AWV on the P–δ curves of NP-ECC-BRs

In a typical P–δ curve of a thin-plate PVA-ECC s specimen, the flexural loads will fluctuate and increase slowly with the increase in deflection after the first crack appears during loading, as shown in Figure 4. Through extensive experimental observation, it was found that the volatility (frequency and value of fluctuation) characteristics of the P–δ curve generally reflected the formation and propagation processes of cracking. That is to say that the number of downward fluctuations of the load at the P–δ curve approximately reflects the number of cracks forming in the specimens, and the levels of load reduction approximately reflect the instantaneous and unstable propagation degree of cracks during the process of cracks from their appearance to maintaining stability. 

**Figure 5:**
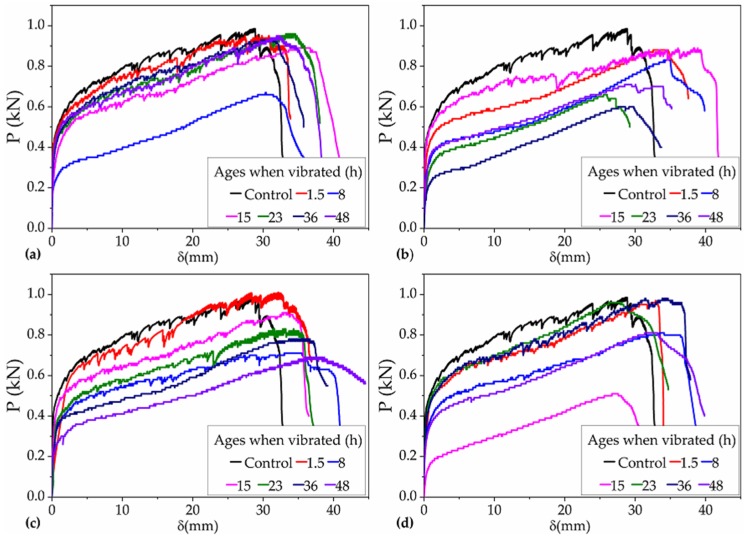
P–δ curves of all the 24 groups of specimens under the operating conditions for Var. 1 with frequencies of (**a**) 2 Hz, (**b**) 3 Hz, (**c**) 4 Hz, and (**d**) 5 Hz and that of the control group.

According to the collected data, the P–δ curves of all the 24 groups of PVA-ECC specimens were obtained, as shown in Figure 5.

It can be seen in Figure 5 that the volatility of the P–δ curves of most of the vibrated groups was not as obvious as that of the control group, indicating that the TRVs that occurred during setting periods affected the multi-cracking characteristics of NP-ECC-BRs to some extent. It can also be seen in Figure 5 that significant hardening segments appeared in the P–δ curves of all the specimens under the operating conditions for Var. 1. In the hardening segments, the flexural loads of the specimens increased slowly with the increase in the corresponding deflection, and the segments presented significant deformation-hardening or strain-hardening characteristics, indicating that vibrations that occurred during different setting periods have no significantly negative impact on the inherent strain-hardening behavior of PVA-ECCs under the operating conditions for Var. 1.The above results are consistent with the tensile stress–strain curves of NP-ECC-BRs that were obtained under the same experimental and parametric conditions in [29].

#### 3.1.2. Effects of AWV on the Flexural Properties of NP-ECC-BRs

**Figure 6:**
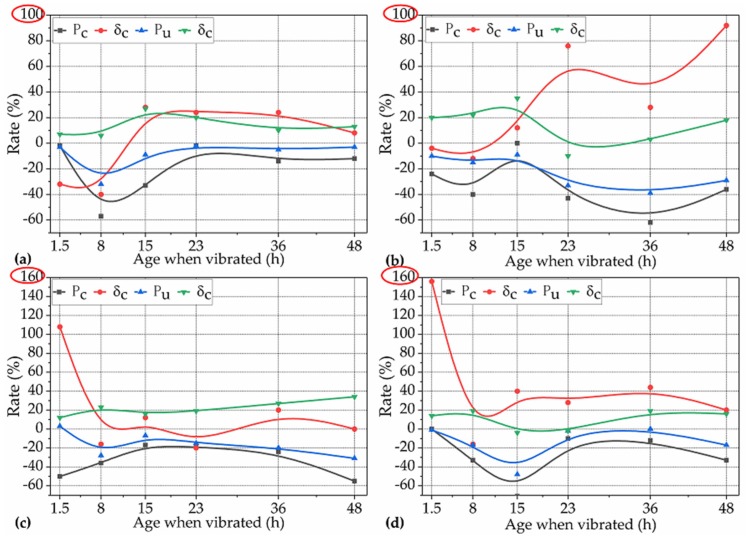
Rates of P_c_, δ_c_, P_u_, and δ_u_ for the 24 groups of specimens over the corresponding control averages under the operating conditions for Var. 1 with frequencies of (**a**) 2 Hz, (**b**) 3 Hz, (**c**) 4 Hz, and (**d**) 5 Hz.

In this subsection, the question of to what extent the TRVs that occurred during different setting periods affected the P_c_, δ_c_, P_u_, and δ_u_ of NP-ECC-BRs was investigated and determined quantitively. The rates of the P_c_, δ_c_, P_u_, and δ_u_ of the groups that were vibrated under the operating conditions for Var. 1 over the corresponding control winsorized means are shown in Figure 6.

Effects of AWV on the cracking load (P_c_) of NP-ECC-BRs. It can be seen in Figure 6a–d (black lines) that the P_c_ of each group of specimens decreased by 2%–71% over the control average under the operating conditions for Var. 1, indicating that the effects of AWV on the P_c_ of NP-ECC-BRs are significantly negative throughout the three setting periods. Furthermore, when the specimens were subjected to frequencies of 2 and 5 Hz, the AWV that resulted in the most negative effects for P_c_ occurred at ages of 8, 15, or 23 h which were within the setting period (the period between the initial and final set), as shown in Figure 6a,d. However, when the specimens were subjected to the frequencies of 3 and 4 Hz, the AWV that resulted in the most negative effects for P_c_ occurred at the ages of 36 or 48 h which were after the final set, as shown in Figure 6b,c.

Effects of AWV on the extreme flexural load (P_u_) of NP-ECC-BRs. It can be seen in Figure 6 that the P_u_ (blue lines) of NP-ECC-BRs specimens decreased by 1%–39% over the control average under the operating conditions for Var. 1, which shows a similar impact trend of to that of P_c_. Moreover, it also can be seen in Figure 6 that the rates of P_u_ and P_c_ increase or decrease in harmony with the increase in the AWV, and the most negative effect for P_u_ also occurred at the same AWV as those of P_c_ under the same vibration frequency (2−5 Hz).

Effects of AWV on the extreme flexural deformation (δ_u_) of NP-ECC-BRs. The effects of AWV on the extreme deformation of NP-ECC-BRs specimens were very different to that of the load-bearing properties (P_c_, P_u_). The effects of the AWV on the δ_u_ of almost all the NP-ECC-BR groups (except for the group F3–23–5, which decreased by 2% and can be ignored) tended to be insignificantly positive, and for most of them, the positive impact degrees were within 20%, as shown in Figure 6a–d (green lines).

Effects of AWV on the cracking deformation (δ_c_) of NP-ECC-BRs. Before the initial set and under relatively lower magnitudes of vibration frequency (2 and 3 Hz), it can be seen in Figure 6a,b (red lines) that the effects of AWV on the δ_c_ of all the NP-ECC-BR groups tended to be insignificantly negative (within 20% reduction). On the contrary, the effects were significantly positive (more than 20% growth) on the δ_c_ of most of the vibrated groups under a relatively higher vibration frequency (4 and 5 Hz) during the same setting period, as shown in Figure 6c,d.

In summary, these above results indicate that the effects of TRVs that occurred during different setting periods on the cracking and extreme load-bearing capacity of NP-ECC-BRs tend to be significantly negative under the combination of different frequencies ranging from 2 to 5 Hz and a constant duration of 5 h. On the contrary, the effects of AWV on the extreme flexural deformation of NP-ECC-BRs generally tended to be insignificantly positive. In addition, the effects of AWV on the cracking deformation of newly placed PVA-ECCs varied according to the corresponding vibration frequency that the specimens suffered when TRVs occurred before the initial set.

### 3.2. Effects of Duration of Vibration (DV) on the Flexural Properties of NP-ECC-BRs

In this section, to determine the effects of DV on the flexural properties of NP-ECC-BRs when vibrations only occurred during the setting period (the period between the initial and final set, where the penetration resistance was within the scope of 3.5 to 28 MPa), a total of 144 (16 groups) thin-plate PVA-ECC specimens were tested by a four-point flexural test after the specimens were vibrated under the operating conditions for Var. 2. 

#### 3.2.1. Effects of DV on the P–δ Curves of NP-ECC-BRs

**Figure 7:**
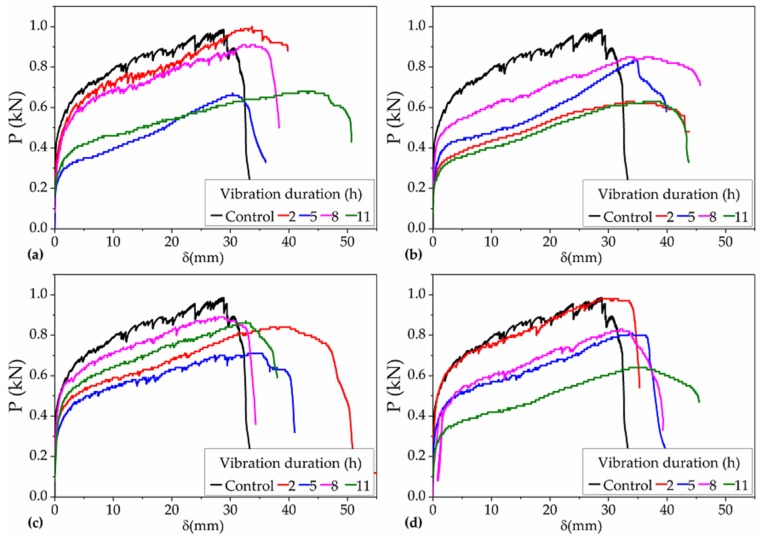
P–δ curves of all the 16 groups of specimens under the operating conditions for Var. 2 with frequencies of (**a**) 2 Hz, (**b**) 3 Hz, (**c**) 4 Hz, and (**d**) 5 Hz and that of the control group.

According to the collected data, the P–δ curves of all the 16 groups of PVA-ECC specimens were obtained, as shown in Figure 7.

It can be seen in Figure 7 that the volatility of the P–δ curves of most of the vibrated groups was not so obvious compared to that of the control group, indicating that there is some extent of negative effect on the multi-cracking characteristics of NP-ECC-BR specimens when the specimens were vibrated during the setting with different DVs. Even so, it also can be seen in Figure 7 that significant hardening segments appeared in the P–δ curves of all the 16 groups of specimens when they were subjected to TRVs with a duration completely covering the setting period. In this section, the longest DV was 11 h, the TRVs began at the age of 8 h, and the time of the final set was 23.8 h. Therefore, for the series of F2,3,4,5–8–11, the duration of 11 h almost covered the whole setting period.

Combined with the results in Subsection 3.1.1 and the results of our previous work in [29], it can be further concluded that the effects of TRVs are not significant, but to some extent there are still negative effects on the strain-hardening behaviors of NP-ECC-BRs within the limits in these studies. On this point, this result was also consistent with the previously found effects of TRVs on the compressive behaviors of NP-C-BRs [17,18,19,20,21].

#### 3.2.2. Effects of DV on the Flexural Properties of NP-ECC-BRs

**Figure 8:**
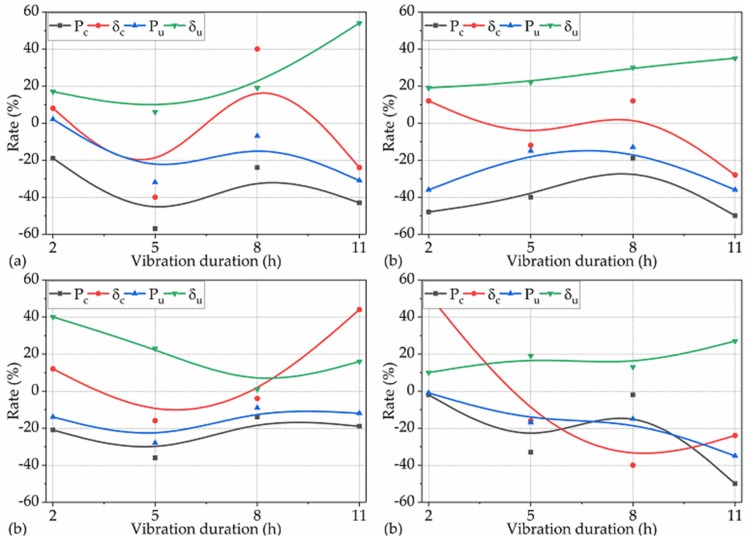
Rates of the P_c_, δ_c_, P_u_, and δ_u_ of the 16 groups of specimens over the corresponding control averages under the operating conditions for Var. 2 with frequencies of (**a**) 2 Hz, (**b**) 3 Hz, (**c**) 4 Hz, and (**d**) 5 Hz.

In this subsection, we investigate and quantitively characterize the extent to which the DV during the setting period affected the P_c_, δ_c_, P_u_, and δ_u_ of NP-ECC-BRs. The rates of the P_c_, δ_c_, P_u_, and δ_u_ of the groups that were vibrated under the operating conditions for Var. 2 over the corresponding control winsorized means are shown in Figure 8.

The effects of DV on the P_c_ of NP-ECC-BRs under the operating conditions for Var. 2 are examined. Similar to the P_c_ obtained under the operating conditions for Var. 1, the effects of DV on the P_c_ of all the 16 groups of NP-ECC-BRs tended to be significantly negative under the operating conditions for Var. 2, decreasing by 19%–57% over the control average, as shown in Figure 8a–d (black lines). Furthermore, P_c_ did not change significantly with the increase in DV under the imposed vibration frequencies of 2, 3, and 4 Hz, as shown in Figure 8a–c, while it presented an obvious reduction trend under the imposed vibration frequency of 5 Hz, as shown in Figure 8d. Based on these results and the results obtained in Section 3.1.2, it could be further concluded that the effects of TRVs on the cracking load-bearing capacity of NP-ECC-BRs tended to be significantly negative, while it was not sensitive to increases in the DV when vibrations occurred during the setting period and when the vibration frequencies were lower than 5.0 Hz. 

The effects of DV on the P_u_ of NP-ECC-BRs under the operating conditions for Var. 2 were examined. Likewise, a similar impact trend was presented for P_u_ compared to P_c_ under the operating conditions for Var. 2. The difference was that the impact degree of DV on P_u_ was less obvious than that of P_c_ in general, as shown in Figure 8a–d (blue lines).

The effects of DV on the δ_u_ of NP-ECC-BRs under the operating conditions for Var. 2 were examined. However, the effects of DV on the δ_u_ of all the 16 groups of specimens was positive, and for most of them, the positive impact degrees were above 20% under the operating conditions for Var. 2, as shown in Figure 8a–d (green lines). Moreover, δ_u_ increased with the increasing of the DV under the imposed vibration frequencies of 2, 3, and 5 Hz, as shown in Figure 8a–c, but it decreased under the imposed vibration frequency of 4 Hz, as shown in Figure 8d.

The effects of DV on the δ_c_ of NP-ECC-BRs under the operating conditions for Var. 2 were examined. It can be seen in Figure 8a–d (red lines) that the effects, regardless of the impact trend, polarity, or degree of DV on the δ_c_ of the specimens, varied according to the corresponding frequencies that the specimens were subjected to.

These above results indicate that the effects of DV on the cracking and extreme load-bearing capacity of NP-ECC-BRs were significantly negative (19%–57% reduction for most of them) under the operating conditions for Var. 2. By contrast, the effects of DV on the extreme flexural deformation were significantly positive, increasing by over 20% under the operating conditions for Var. 2. Moreover, the results in Figure 8 also indicate that the longer the DVs, the higher the extreme flexural deformation capacity of NP-ECC-BRs when TRVs occurred only during the setting period. Additionally, the effects of DV on the cracking deformation of NP-ECC-BRs seem to vary according to the corresponding vibration frequencies that the specimens experienced, regardless of the vibration variables of AWV and DV.

These above results regarding the flexural load-bearing capacity obtained in this section was similar to that of the ultimate tensile strength of NP-ECC-BRs in [29], as well as the compressive strength, bond strength, splitting tensile strength, and flexural strength of NP-C-BRs when vibrations occurred only during the setting period [22,23,24]. The results in [22,23,24,29] showed that the TRVs that occurred only during the setting periods affected the performance of NP-C-BRs or NP-ECC-BRs to some extent which should not be ignored. 

However, these above results concerning the extreme flexural deformation were just opposite to the tensile deformation properties of NP-ECC-BRs when TRVs occurred only during the setting period [29]. The result in [29] showed that the effects of DVs ranging from 2 to 11 h on the extreme tensile deformation capacity of NP-ECC-BRs tended to be negative overall.

### 3.3. Effects of Vibration Frequency on the Flexural Properties of NP-ECC-BRs

**Figure 9:**
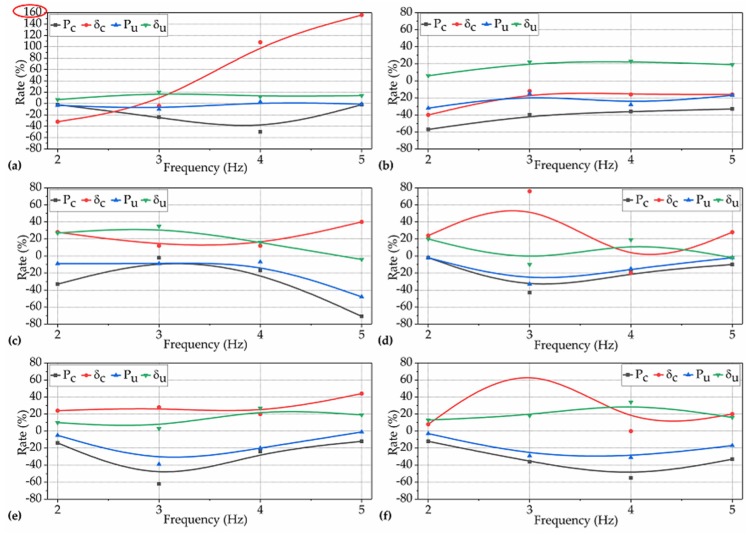
The rates of flexural properties with different vibration frequencies over the control averages under the operating conditions for Var. 1 at ages of (**a**) 1.5 h, (**b**) 8 h, (**c**) 15 h, (**d**) 23 h, (**e**) 36 h, and (**f**) 48 h.

**Figure 10:**
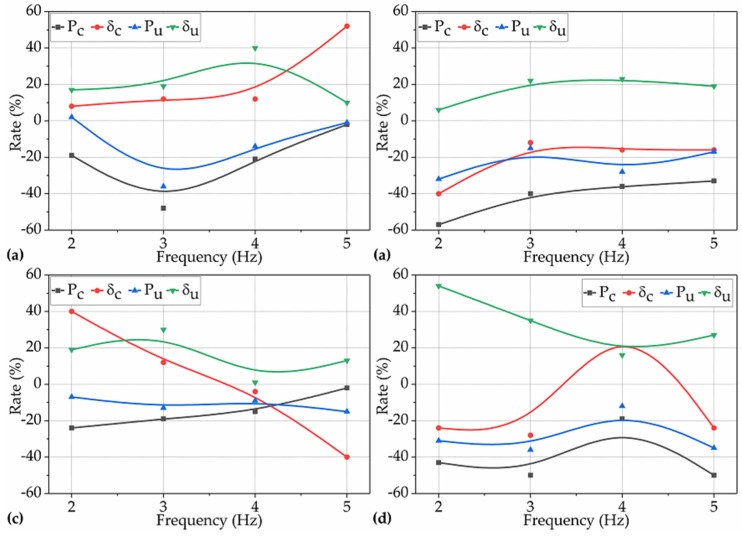
The effects of vibration frequency on the flexural properties of newly placed PVA-ECC bridge repairs (NP-ECC-BRs) under the operating conditions for Var. 2 with durations of (**a**) 2 h, (**b**) 5 h, (**c**) 8 h, and (**d**) 11 h.

The results in Section 3.1 and Section 3.2 show that effects of TRVs on the flexural properties of NP-ECC-BRs are related to the corresponding vibration frequency. Accordingly, to further quantitatively investigate the flexural properties of NP-ECC-BRs when imposing different vibration frequencies, the effects of vibration frequency on the flexural properties of NP-ECC-BRs are analyzed in this section. The rates of flexural properties of the specimens over the control averages under the operating conditions for Var. 1 and Var. 2 are shown in Figure 9 and Figure 10, respectively.

#### 3.3.1. Effects of Vibration Frequency on the Flexural Properties of NP-ECC-BRs under the Operating Conditions for Var. 1

Effects of Vibration frequency on the flexural properties (P_c_, δ_c_, P_u_, and δ_u_) of NP-ECC-BRs when vibrations occurred before the initial set were examined. It can be seen in Figure 9a that the P_c_ of the vibrated specimens changed in an approximately linearly fashion from −2% to −24%, and −50% over the control average with an increase in vibration frequency ranging from 2 to 4 Hz, and this changed up to −2% under the vibration frequency of 5 Hz. It also can be seen in Figure 9a that δ_c_ presented a roughly opposite trend compared with P_c_, which changed in an approximately linearly fashion from −32% to 156% over the control average with an increase in vibration frequency ranging from 2 to 5 Hz. Moreover, the effects of vibration frequency on the extreme flexural properties (P_u_ and δ_u_) were not so obvious compared to those of the cracking properties (P_c_ and δ_c_) when vibrations occurred before the initial set.

Effects of Vibration frequency on the flexural properties (P_c_, δ_c_, P_u_ and δ_u_) of NP-ECC-BRs when vibrations began just after the initial set were examined. When the specimens were vibrated at the age of 8 h, it can be seen in Figure 9b that all the flexural properties increase or decrease in harmony and approximately linearly with the increase in vibration frequency. The differences are that the impact polarity was negative on P_u_, P_c_, and δ_c_, and it was positive on δ_u_. Additionally, the impact degree of TRVs on P_c_ was the largest, indicating that P_c_ was the most sensitive in its response to TRVs during these periods, followed by δ_c_ or P_u_, and then δ_u_.

Effects of Vibration frequency on the flexural properties (P_c_, δ_c_, P_u_ and δ_u_) of NP-ECC-BRs when vibrations occurred after the middle stage of the setting period were examined. When the specimens were vibrated after the age of 15 h, it can be seen in Figure 9c–f that the effects of TRVs on the load-bearing properties (P_c_ and P_u_) of the specimens was negative throughout the scope of imposed vibration frequencies in this study. However, it was positive on the deformation properties (δ_c_ and δ_u_). Figure 9c–f also show that the flexural properties of most of the vibrated groups declined, in general, with the increase in imposed vibration frequency. Moreover, regarding the impact degree (regardless of polarity), the cracking properties (the P_c_ and δ_c_ of most of the specimens presented a reduction or growth above 20%) were more sensitive to the imposed vibration frequency than those of the extreme flexural properties (the P_u_ and δ_u_ of all the specimens presented a reduction or growth within 20%).

#### 3.3.2. Effects of Vibration Frequency on the Flexural Properties of NP-ECC-BRs under the Operating Conditions for Var. 2

As described in Figure 9b and shown in Figure 10b, the effects of imposed vibration frequency on the flexural properties (P_u_, δ_u_, P_c_, and δ_c_) of NP-ECC-BRs specimens is that they increase or decrease in an approximately linearly fashion with the increase in vibration frequency with the constant duration of 5 h during the setting period. However, the results in Figure 10a,c,d show that when subjected to durations of 2, 8, and 11 h during the setting period, the effects of imposed vibration frequency on the deformation properties (δ_c_ and δ_u_) of some of the vibrated groups were obviously different to those with the constant duration of 5 h. The obvious observed differences can be described as follows: (1) with a duration of 2 h, the effects of TRVs on the deformation properties (δ_c_ and δ_u_) of the specimens were positive, and they generally increased with an increase in vibration frequency, as shown in Figure 10a; (2) with a duration of 8 h, the rate of the δ_c_ of the vibrated PVA-ECC specimens decreased approximately linearly from 40% to − 40% over the control average, as shown in Figure 10c; (3) when subjected to a duration of 11 h, the δ_c_ of the vibrated specimens increased by 44% over the control group under the vibration frequency of 4 Hz.

### 3.4. Explanations and Recommendations

Regarding the reduction of load-bearing capacity of NP-ECC-BRs under operating conditions for Var. 1 and 2, the following explanations can be made: before the initial set, continuous TRVs will lead to some extent of bleeding to the cement mixture [10], and the stronger the magnitudes of vibration frequency, the greater the amount of bleeding this would lead to; after the initial set, low-density C-S-H gels in the cement matrix gradually are transferred to high-density C-S-H gels and agglomerate into larger C-S-H particles [11,12], which gradually form the solid skeleton of the cement matrix, and TRVs during this period are likely to disturb the connection of the solid skeleton in the cement matrix or even damage the bond of C-S-H particles and C-S-H gels [13]; these above effects demonstrate a great potential for decreasing the cracking and extreme flexural load-bearing capacity of the cement matrix. According to the fiber-bridge constitutive law of engineered cementitious composites (ECCs) and its design principle [35,36], relatively lower cracking strength is beneficial for ECCs to express their bridging effect between PVA fibers and cement matrix and to exhibit larger deformation [37,38]. Therefore, TRVs could result in a negative effect on the load-bearing capacity of NP-ECC-BRs while resulting in a positive effect on the flexural deformation capacity of NP-ECC-BRs.

With regard of the reduction of load-bearing capacity of NP-ECC-BRs under operating conditions for Var. 1 and 2, the authors recommend that (a) in the design of PVA-ECC bridge repairs, engineers should try to improve the design strength of PVA-ECCs, especially its early-age strengths, such as adding an appropriate amount of silica fume and early-strength agents, adopting well-graded silicon sand and high-strength cement; and (b) during the repairing of concrete bridge using PVA-ECCs, constructors should restrict the traffic of heavy-duty trucks and medium-size vehicles in large- and medium-spans of concrete bridges, especially during the period between the initial and final set of PVA-ECC repairs.

## 4. Conclusions

(1) The effects of traffic vibrations are not determinantal, but to some extent, there are negative effects on the strain-hardening behavior of newly placed PVA-ECC bridge repairs within the limits in this investigation.
(2) The effects of traffic vibrations on the cracking and extreme load-bearing capacity of newly placed PVA-ECC bridge repairs were significantly negative (above 20% reduction) for most of the vibrated groups in this investigation. By contrast, the effects were significantly positive (above 20% growth) on the extreme flexural deformation of newly placed PVA-ECC bridge repairs. Moreover, the longer the durations of vibration, the higher the extreme flexural deformation capacity of newly placed PVA-ECC bridge repairs, generally.
(3) The effects of traffic vibrations on the cracking deformation of newly placed PVA-ECCs varied according to the corresponding vibration frequency that the specimens suffered.
(4) Based on the results obtained, we concluded that the effects of traffic vibrations on the flexural deformation of PVA-ECC bridge repairs should not be a cause for concern, however, serious consideration should be given to the associated reduction of load-bearing capacity.
(5) The authors recommend that (a) in the design of PVA-ECC bridge repairs, engineers should try to improve the design strength of PVA-ECCs, especially its early-age strengths, such as adding an appropriate amount of silica fume and early-strength agents, adopting well-graded silicon sand and high-strength cement; and (b) during the repairing of concrete bridges using PVA-ECCs, constructors should restrict the traffic of heavy-duty trucks and medium-size vehicles in large- and medium-spans of concrete bridge, especially during the period between the initial and final set of PVA-ECC repairs.

## Figures and Tables

**Table 1:** Source information of materials (Product Model, Manufacturer, City, Country).

Materials	Product Model	Manufacturer	City	Country
Portland cement	P∙O 42.5 R ^a^	Ji Dong Cement	Hohhot	China
Fly ash	Class–І ^b^	Ordos Thermal Power Plant	Ordos	China
Silicon sand	High-quality	Togtoh Silicon Sand	Hohhot	China
Polyvinyl alcohol (PVA) fiber	K–II	Kuraray	–	Japan
High-efficiency water reducing Agent (HEWRA)	3301E	Sika Construction and Building Materials	Dalian	China
High-efficiency defoamer (HED)	JXPT–1206	Jinliangbo Technology	Beijing	China
Viscosity modifying agent (VMA)	MK–100000S	Chuangyao Biotechnology	Jinan	China

^a^ The “P∙O 42.5 R” Portland cement represented the compressive and flexural strengths of tested samples that were higher than 22.0 and 4.0 MPa after 3 days’ curing, and were higher than 42.5 and 4.0 MPa after 28 days’ curing according to Chinese National Standards GB175–2007 [30]. ^b^ The main physical property indexes of “Class-I” fly ash must meet the requirements of fineness ≤12.0% (residue after being screened with a 45 μm square mesh sieve), water demand ratio ≤95%, and loss on ignition ≤5% according to Chinese National Standards GB/T1596–2017 [31].

**Table 2:** Basic physical indexes of Portland cement.

Setting Time (h)		Water Requirement of Normal Consistency (%)	Flexural Strength (MPa)		Compressive Strength (MPa)	
Initial	Final	26.93	3 days	28 days	3 days	28 days
1.95	2.98		5.82	8.14	28.92	47.64

**Table 3:** Chemical compositions of Portland cement (%).

Al_2_O_3_	SiO_2_	CaO	Fe_2_O_3_	MgO	SO_3_	Loss on Ignition
7.2	23.4	55.0	3.0	2.2	2.9	2.9

**Table 4:** Chemical compositions of fly ash (%).

SiO_2_	Al_2_O_3_	CaO	Fe_2_O_3_	CO_2_	MgO	SO_3_	K_2_O	Na_2_O	TiO_2_	SrO	Others
40.3	18.2	18.1	8.5	5.2	2.3	2.1	1.8	1.3	1.0	0.7	0.6

**Table 5:** Physical properties of polyvinyl alcohol (PVA) fiber.

Fineness (dtex)	Density (g/cm^3^)	Diameter (μm)	Elongation (%)	Tensile Strength (MPa)	Length (mm)	Young’s Modulus (GPa)
15	1.3	40	6	1600	12	40

**Table 6:** Mixture proportions of the material (kg/m^3^).

Portland Cement	Silicon Sand	Fly Ash	Water	HEWRA	HED	VMA	PVA Fiber
254	457	1016	304	15.2	2.6	0.6	26

**Table 7:** The ages at which the specimens were vibrated and their corresponding setting periods

Setting Periods	Before the Initial Set	During the Setting Period	After the Final Set
Penetration Resistance (MPa)	≤3.5	3.5–28.0	≥28.0
Setting Time (h)	0–7.6	7.6–23.8	≥23.8
Ages When Vibrated (h)	1.5	8, 15, 23	36, 48

**Table 8:** Specimen design.

Specimen Group	Operating Conditions	Vibration Frequency (Hz)	Age When Vibrated (h)	Duration of Vibration (h)	Vibration Amplitude (mm)	Number of Specimens	Age When Tested (Days)
Control	Var. 1	–	–	–	–	9	28
F2–1.5–5		2	1.5	5	5	9	28
F2–8–5		2	8	5	5	9	28
F2–15–5		2	15	5	5	9	28
F2–23–5		2	23	5	5	9	28
F2–36–5		2	36	5	5	9	28
F2–48–5		2	48	5	5	9	28
F3-1.5-5		3	1.5	5	5	9	28
F3-8-5		3	8	5	5	9	28
F3-15-5		3	15	5	5	9	28
F3-23-5		3	23	5	5	9	28
F3-36-5		3	36	5	5	9	28
F3-48-5		3	48	5	5	9	28
F4–1.5–5		4	1.5	5	5	9	28
F4–8–5		4	8	5	5	9	28
F4–15–5		4	15	5	5	9	28
F4–23–5		4	23	5	5	9	28
F4–36–5		4	36	5	5	9	28
F4–48–5		4	48	5	5	9	28
F5–1.5–5		5	1.5	5	5	9	28
F5–8–5		5	8	5	5	9	28
F5-15-5		5	15	5	5	9	28
F5–23–5		5	23	5	5	9	28
F5–36–5		5	36	5	5	9	28
F5–48–5		5	48	5	5	9	28
F2–8–2	Var. 2	2	8	2	5	9	28
F2–8–5		2	8	5	5	9	28
F2–8–8		2	8	8	5	9	28
F2–8-11		2	8	11	5	9	28
F3–8–2		3	8	2	5	9	28
F3–8–5		3	8	5	5	9	28
F3–8–8		3	8	8	5	9	28
F3–8–11		3	8	11	5	9	28
F4–8–2		4	8	2	5	9	28
F4–8–5		4	8	5	5	9	28
F4–8–8		4	8	8	5	9	28
F4–8–11		4	8	11	5	9	28
F5–8–2		5	8	2	5	9	28
F5–8–5		5	8	5	5	9	28
F5–8–8		5	8	8	5	9	28
F5–8–11		5	8	11	5	9	28

**Note:** “F2–1.5–5” means that the corresponding group of specimens was vibrated at the age of 1.5 h under the combination of a duration of 5.0 h and a vibration frequency of 2 Hz. A similar naming format is used to indicate the test conditions for the other groups.

**Table 9:** Winsorized means of the cracking load (P_c_), mid-span deflection (δ_c_), extreme flexural load (P_u_), and mid-span deflection (δ_u_) of all 37 groups of polyvinyl alcohol fiber reinforced engineering cementitious composite (PVA-ECC) specimens.

Specimen Group	Operating Conditions	P_c_ (kN)	ΔP_c_ (%)	δ_c_ (mm)	Δδ_c_ (%)	P_u_ (MPa)	ΔP_u_ (%)	δ_u_ (mm)	Δδ_u_ (%)
Control	Var. 1	0.42		0.25		0.98		28.8	
F2–1.5–5		0.41	–2	0.17	−32	0.95	−3	30.8	7
F2–8.0–5		0.18	−57	0.15	−40	0.67	−32	30.4	6
F2–15.0–5		0.28	−33	0.32	28	0.89	−9	36.7	27
F2–23.0–5		0.41	−2	0.31	24	0.96	−2	34.6	20
F2–36.0–5		0.36	−14	0.31	24	0.93	−5	31.7	10
F2–48.0–5		0.37	−12	0.27	8	0.95	−3	32.4	13
F3–1.5–5		0.32	−24	0.24	−4	0.88	−10	34.7	20
F3–8.0–5		0.25	−40	0.22	−12	0.83	−15	35	22
F3–15.0–5		0.41	−2	0.28	12	0.89	−9	39	35
F3–23.0–5		0.24	−43	0.44	76	0.66	−33	26	−10
F3–36.0–5		0.16	−62	0.32	28	0.6	−39	29.8	3
F3–48.0–5		0.27	−36	0.48	92	0.7	−29	34	18
F4–1.5–5		0.21	−50	0.52	108	1.01	3	32.25	12
F4–8.0–5		0.27	−36	0.21	−16	0.71	−28	35.5	23
F4–15.0–5		0.35	−17	0.28	12	0.91	−7	33.4	16
F4–23.0–5		0.19	−19	0.2	−20	0.83	−15	34.3	19
F4–36.0–5		0.32	−24	0.3	20	0.78	−20	36.6	27
F4–48.0–5		0.2	−55	0.25	0	0.68	−31	38.5	34
F5–1.5–5		0.41	−2	0.64	156	0.97	−1	32.8	14
F5–8.0–5		0.28	−33	0.21	−16	0.81	−17	34.2	19
F5–15.0–5		0.12	−71	0.35	40	0.51	−48	27.6	−4
F5–23.0–5		0.38	−10	0.32	28	0.96	−2	28.2	−2
F5–36.0–5		0.37	−12	0.36	44	0.97	−1	34.2	19
F5–48.0–5		0.28	−33	0.3	20	0.81	−17	33.3	16
F2–8.0–2	Var. 2	0.34	−19	0.27	8	1	2	33.7	17
F2–8.0–5		0.18	−57	0.15	−40	0.67	−32	30.4	6
F2–8.0–8		0.32	−24	0.35	40	0.91	−7	34.3	19
F2–8.0–11		0.24	−43	0.19	−24	0.68	−31	44.4	54
F3–8.0–2		0.22	−48	0.28	12	0.63	−36	34.4	19
F3–8.0–5		0.25	−40	0.22	−12	0.83	−15	35	22
F3–8.0–8		0.34	−19	0.28	12	0.85	−13	37.5	30
F3–8.0–11		0.21	−50	0.18	−28	0.63	−36	38.9	35
F4–8.0–2		0.33	−21	0.28	12	0.84	−14	40.2	40
F4–8.0–5		0.27	−36	0.21	−16	0.71	−28	35.5	23
F4–8.0–8		0.35	−15	0.24	−4	0.89	−9	29	1
F4–8.0–11		0.34	−19	0.36	44	0.86	−12	33.5	16
F5–8.0–2		0.41	−2	0.38	52	0.97	−1	31.6	10
F5–8.0–5		0.28	−33	0.21	−16	0.81	−17	34.2	19
F5–8.0–8		0.41	−2	0.15	−40	0.83	−15	32.4	13
F5–8.0–11		0.21	−50	0.19	−24	0.64	−35	36.5	27
